# How to Define High-Flow Arteriovenous Fistula? Comment on Kim et al. Hemodynamic Adaptation and Cardiac Effects of High-Flow Arteriovenous Access in Hemodialysis Patients: A Prospective Study. *J. Clin. Med.* 2025, *14*, 4556

**DOI:** 10.3390/jcm15010114

**Published:** 2025-12-24

**Authors:** Jan Malik, Anna Valerianova, Kristina Buryskova Salajova, Pavel Michalek

**Affiliations:** General University Hospital and First Faculty of Medicine, Charles University, 12808 Prague, Czech Republic; anna.valerianova@vfn.cz (A.V.); kristina.buryskova@vfn.cz (K.B.S.); pavel.michalek@vfn.cz (P.M.)

We have read the manuscript by Yaeni Kim et al. in the recent volume of the *Journal of Clinical Medicine* with great interest [1]. The authors studied two groups of haemodialysis patients based on the ratio of the arteriovenous fistula flow (Qa) to the cardiac output (CO), specifically those with rations less than or greater than 0.3. Patients who had a Qa/CO ratio > 0.3 had upper-arm arteriovenous fistula (AVF) more frequently and had a higher risk of developing high-output heart failure within a year even though they showed a lower(!) Qa at inclusion.

AVFs with a high Qa are known to contribute to the development of high-output heart failure (HOHF), which is usually described by the presence of heart failure signs and symptoms and by the cardiac index > 3.5–4.0 L/min/m^2^ [2]. Several surgical techniques have been published that enable Qa reduction [3]. Generally, three definitions of high-flow AVFs have been used in the literature: 1. Qa/CO > 25–30%; 2. Qa > 1500–2000 mL/min; 3. Presence of HOHF [4,5,6]. Patients with any of the three definitions were selected to undergo flow-reducing surgery provided that they had heart failure symptoms (with 1 exception [5]).

It would be reasonable to think that all three definitions represent equal or similar populations. However, the study by Yaeni Kim et al. [1] provided different results: patients with Qa/CO > 0.3 had lower CO. This statement contradicts the general intuition that the surgical creation of an AVF leads to a sudden fall of the systemic vascular resistance and an increase in the venous return, which is followed by an increase in cardiac output. The next step is a further decrease in peripheral vascular resistance to ensure organ perfusion and, in turn, an even higher CO increase. Indeed, patients with HOHF had the lowest values of systemic vascular resistance [7]. Yaeni Kim et al. included prevalent patients with a mean dialysis vintage above 65 months, so the cardiovascular system of the included patients was given enough time to adapt to AVF creation. At first, we were sceptical regarding these findings, so we analysed the included instances of CZecking Heart Failure in the study of Chronic Kidney Disease [7]. We obtained similar results to Yeani Kim et al.—see Table 1 for details.

Our group is larger and differs from that of Yaeni Kim et al. in having a shorter dialysis vintage and in having practically equal total CO in both groups. Interestingly, the effective CO was higher in patients with a lower Qa/CO ratio, despite the estimated systemic vascular resistance being lower. In other words, in patients with a higher Qa/CO ratio, the higher systemic vascular resistance did not allow for an adequate increase in cardiac output, and resulting tissue/organ perfusion was lower. When we estimated differences in the systemic vascular resistance, explored in the manuscript by Yaeni Kim et al., we obtained a similar difference.

Altogether, there are at least two different phenotypes of high-flow AVFs. If we use the definition involving the Qa/CO ratio, we include more patients with insufficient CO increase, probably due to heart failure or its structural and functional abnormalities. Such patients probably suffer from so-called forward heart failure symptoms, known in the non-haemodialysis heart failure population, but future studies should help prove this observation. Thanks to the work of Yaeni Kim et al., we now know that these patients are at higher risk of classic HOHF development. We can only speculate that it is due to left ventricular dilatation and increased filling pressures as an adaptive mechanism at the cost of lung congestion (the backward heart failure symptom). Another question is whether these adaptive changes become irreversible despite the surgical reduction or ligation of the AVF.

We believe that Yaeni Kim et al. can offer ideas regarding the definition of a high-flow AVF. Another question we have is why they also presented a sum of (the total) CO and Qa.

## Figures and Tables

**Table 1:** Characteristics of patients according to Qa/CO ratio.

Variable	Qa/CO < 0.3	Qa/CO > 0.3	*p*-Value
N	202	45	
Age (years)	70.3 (59.5–77.3)	59.6 (49.2–73.8)	0.003
Dialysis vintage (months)	28.0 (12.0–69.0)	51.5 (22.5–114.0)	0.005
NTproBNP (ng/L)	4772 (1899–13,966)	7197 (3210–35,000)	0.049
Qa (mL/min)	1000 (780–1280)	2300 (1550–2700)	<10^−5^
Body mass index (kg/m^2^)	25.6 (22.5–29.8)	25.4 (22.5–30.1)	0.91
Systolic blood pressure (mmHg)	135 (120–151)	134 (115–151)	0.64
Diastolic blood pressure (mmHg)	74 (64–82)	78 (68–88)	0.05
Cardiac output (L/min)	6.0 (5.0–7.0)	6.1 (5.0–7.1)	0.97
Effective cardiac output (L/min)	5.0 (4.0–5.9)	3.7 (3.0–4.8)	<10^−5^
Systemic vascular resistance (Wood units)	17.7 (13.8–22.2)	26.1 (20.5–30.7)	<10^−5^

Legend: Qa = arteriovenous fistula flow volume; CO = cardiac output; effective cardiac output = difference between the total CO and Qa. Data are presented as median (interquartile range) and compared using Mann–Whitney test.

## Abbreviations

The following abbreviations are used in this manuscript:

Qa	Arteriovenous fistula flow volume
CO	Cardiac output
HOHF	High-output heart failure
